# Diagnosis of High-Grade Osteosarcoma by Radiology and Cytology: A Retrospective Study of 52 Cases

**DOI:** 10.1080/13577140410001679239

**Published:** 2004-03

**Authors:** Veli Söderlund, Lambert Skoog, Krishnan K. Unni, Franco Bertoni, Otte Brosjö, Andris Kreicbergs

**Affiliations:** 1 Department of Radiology Karolinska Hospital Stockholm S-171 76 Sweden; 2 Department of Pathology and Cytology Karolinska Hospital Stockholm S-171 76 Sweden; 3 Department of Orthopaedics Karolinska Hospital Stockholm S-171 76 Sweden; 4 Department of Laboratory Medicine and Pathology Mayo Clinic Rochester MN 55905 USA; 5 Instituto Orthopedico Rizzoli Anatomia Patologica Bologna 40136 Italy

## Abstract

The diagnostic value of combined radiology and fine needle aspiration cytology (FNAC) was retrospectively assessed in a consecutive series of 52 patients with high-grade osteosarcoma. The series was divided into typical and atypical osteosarcomas according to radiological features and site. Thirty-two of 33 radiologically typical osteosarcoma cases were correctly diagnosed by cytology; one lesion was diagnosed as sarcoma NOS. Nineteen osteosarcoma cases were radiographically atypical. Six of these were diagnosed as osteosarcoma and another six as sarcoma NOS. In three cases another type of sarcoma was suggested. One case was falsely classified as benign. FNAC of three cases were non-diagnostic. Overall, the diagnostic difficulties pertained to the radiologically atypical cases. Notably, four of these also posed considerable difficulties in the histopathological assessment prompting external consultation. Our study suggests that open biopsy can be obviated in high-grade osteosarcomas exhibiting typical radiological features, i.e., in two-thirds.


*Sarcoma*, March 2004, *VOL. 8, NO. 1, 31–36*

Taylor & Francis healthsciences

**ORIGINAL ARTICLE**

### Diagnosis of high-grade osteosarcoma by radiology and cytology: a retrospective study of 52 cases

VELI SÖDERLUND[1], LAMBERT SKOOG[2], KRISHNAN K. UNNI[4], FRANCO BERTONI, OTTE BROSJÖ[3] & ANDRIS KREICBERGS[3]

*Departments of [1]Radiology, [2]Pathology and Cytology and [3]Orthopaedics, Karolinska Hospital, S-171 76 Stockholm, Sweden; [4]Department of Laboratory Medicine and Pathology, Mayo Clinic, Rochester, MN 55905, USA; [4]Instituto Orthopedico Rizzoli, Anatomia Patologica, 40136 Bologna, Italy*

**Abstract**
The diagnostic value of combined radiology and fine needle aspiration cytology (FNAC) was retrospectively assessed in a consecutive series of 52 patients with high-grade osteosarcoma. The series was divided into typical and atypical osteosarcomas according to radiological features and site. Thirty-two of 33 radiologically typical osteosarcoma cases were correctly diagnosed by cytology; one lesion was diagnosed as sarcoma NOS. Nineteen osteosarcoma cases were radiographically atypical. Six of these were diagnosed as osteosarcoma and another six as sarcoma NOS. In three cases another type of sarcoma was suggested. One case was falsely classified as benign. FNAC of three cases were non-diagnostic. Overall, the diagnostic difficulties pertained to the radiologically atypical cases. Notably, four of these also posed considerable difficulties in the histopathological assessment prompting external consultation. Our study suggests that open biopsy can be obviated in high-grade osteosarcomas exhibiting typical radiological features, i.e., in two-thirds.

**Key words:** *osteosarcoma, radiology, fine needle aspiration biopsy, compliance*

### Introduction

Histopathology on tumour material obtained through open biopsy is considered the golden standard in the diagnosis of bone tumours. However, it remains an in-patient procedure requiring regional or general anaesthesia. Moreover, it entails a risk of compartmental violation, tumour seeding, infection and occasionally fracture.

Fine needle aspiration biopsy (FNAB), as an alternative to open biopsy in the diagnosis of bone lesions, has been met with scepticism. The main criticism against FNAB is the anticipated difficulty in sampling representative cell material from bone tumours because of tumour heterogeneity.[1–7] We have previously reported on fine needle aspiration cytology (FNAC) in the diagnosis of different bone lesions.[8–10] Given that the procedure is diagnostically valid in bone neoplasia, it may significantly reduce the need of open biopsy.

In this present retrospective study of 52 OS patients, the diagnostic value of a combined radiological and cytological approach was assessed.

### Material and methods

From 1991 through 2000, 52 consecutive patients with high-grade osteosarcoma (OS) were referred to the Karolinska Hospital. All were preoperatively evaluated by radiography, MRI and FNAC.

There were 16 females and 36 males. Mean age was 19 (3–78) years; 41 of 52 patients are still alive, 37 without evidence of disease and four with lung metastases after a mean follow up of 4.0 (1–9) years. Ten patients have died of their disease, one of complications from chemotherapy.

### *Radiology*

The lesions were categorised by the radiologist as typical or atypical osteosarcomas according to radiological appearance and anatomical site.

### *Appearance*

Typical radiological features of osteosarcoma were a combination of bone destruction, irregular, spiculated

Correspondence to: Veli Söderlund, MD, Department of Radiology, Karolinska Hospital, S171 76 Stockholm, Sweden. Tel.: +46-8-5177 4509/2000; Fax +46 8 5177 4583; E-mail: veli.soderlund@ks.se

1357-714X print/1369–1643 © 2004 Taylor & Francis Ltd
DOI: 10.1080/13577140410001679239

periosteal reaction and a soft tissue mass. Cases lacking any one of these three features were classified as atypical.

*Site*

The proximal epi-metaphysis of humerus and tibia, and the distal epi-metaphysis of femur were considered as typical sites for osteosarcoma. Other sites were considered atypical.

*Cytology*

FNAB was carried out as an out-patient procedure at the first visit. As 47 of the patients presented with a palpable mass, only five FNABs had to be done under fluoroscopic guidance. Smears from each aspirate were air-dried and stained with May–Grünwald–Giemsa (MGG). The immediate evaluation using a 'quick' MGG stain occasionally prompted an additional FNAB.

### Diagnosis and treatment

Among 52 patients, 26 underwent preoperative chemotherapy and surgery solely based on radiology and cytology. The diagnosis was confirmed with histopathology of the resected specimen. Among the remaining 26 patients, pre-operative open (17 cases) or core (six cases) biopsy was also done in 23 followed by adjuvant chemotherapy and surgery. Three patients were excluded from chemotherapy because of high age (49–78 years) and therefore only managed by FNAC and wide excision.

To obtain a second opinion on the OS diagnosis as assessed by histopathology of open biopsies, the preoperative slides from the 17 cases were reviewed by an external pathologist (Mayo Clinic, Rochester, USA). The examiner was only informed that they had caused diagnostic difficulties in a larger series of bone lesions from another study. Likewise, second opinion on postoperative slides from the 26 patients, who underwent adjuvant chemotherapy and surgery without prior open biopsy, was sought from external expertise to check the preoperative cytological diagnosis. The review was carried out in co-operation between the Karolinska Hospital and Rizzoli Institute (Bologna, Italy) by pathologists not involved in the clinical management. However, the reviewers were informed about the primary cytological diagnosis. Thus, altogether nine of 52 cases were not subjected to external consultation. These comprised the six preoperative core biopsies with limited tissue material preserved for a multicenter study (Italian and Scandinavian Sarcoma Group osteosarcoma protocol I) and another three postoperative specimens with unequivocal histological features of OS from patients not being given preoperative chemotherapy.

### Results

*Typical osteosarcoma*

Of the 52 OS cases, 33 fulfilled the criteria of being radiographically typical (Fig. 1).

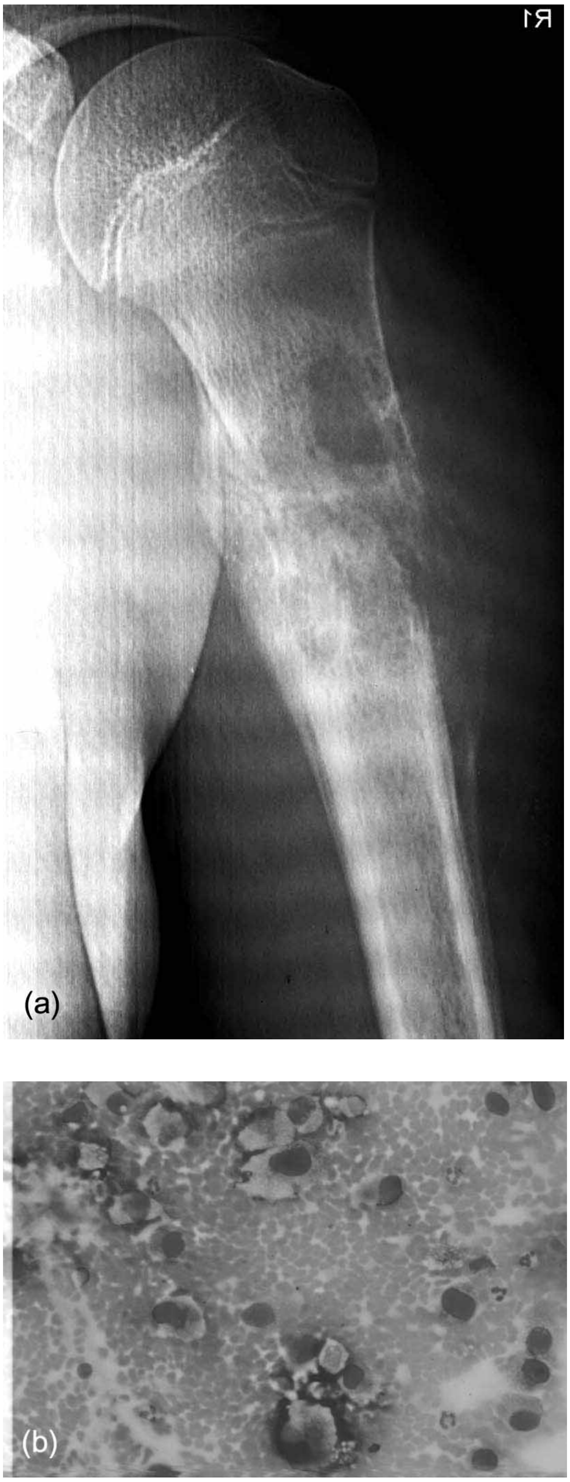

**Fig. 1.** *Radiologically typical osteosarcoma in a 13-year-old boy, with a tumour in the proximal humerus and pathological fracture. Note spiculae and soft tissue mass (a). Cytology showed a mixture of osteoclasts and osteoblasts with marked atypia and osteoid formation characteristic for high-grade osteosarcoma (b).*

Of the 33 typical cases, FNAB was conclusive for OS diagnosis in 32. In 30 cases, cytology showed a mixture of osteoclasts and osteoblasts with marked atypia and osteoid formation. Although two cases lacked osteoid formation, an OS diagnosis was made based on abundant osteoblast-like cells with severe atypia. In one case, the cytological material showed malignant mesenchymal cells, but no osteoid or atypical osteoblasts. Hence, it was classified as sarcoma NOS (Table 1). Altogether, 11 of the 33 typical cases were assessed also by pre-operative open (eight) and core (three) biopsy. Review (Mayo Clinic) of the eight open biopsies, confirmed the OS diagnosis.

Twenty-two of the 33 typical OS cases received adjuvant chemotherapy exclusively based on radiology and cytology. The histopathological review (Rizzoli Institute) of tissue material from the 22 resected specimens consistently confirmed the pre-operative cytological diagnosis.

The cytological diagnoses and radiological assessments are shown in Table 2.

### *Atypical osteosarcoma*

Nineteen OS cases were atypical according to radiological appearance and/or anatomical site (Table 2). The radiological differential diagnoses were one case each of eosinophilic granuloma, chondrosarcoma, malignant fibrous histiocytoma, probable osteosarcoma and osteosarcoma, two cases of osteomyelitis, aneurysmal bone cyst, chondromyxofibroma, giant cell tumour and Ewing's sarcoma and four cases of myeloma or metastasis. The cytological diagnosis of the 19 atypical cases is shown in Table 3. As can be seen, a conclusive OS diagnosis

**Table 1.** *Diagnosis of the 52 osteosarcoma cases according radiology and cytology*

| Osteosarcomas (52) | Cytology |
|---|---|
| Typical, 33 | 32 osteosarcoma<br>1 sarcoma NOS |
| Atypical, 19 | 6 osteosarcoma<br>6 sarcoma NOS<br>3 other sarcoma<br>1 benign<br>3 not diagnostic |

**Table 2.** *Cytological diagnosis in relation to radiological assessment*

| Cytological diagnosis | Number | Typical | Atypical |
|---|---|---|---|
| Osteosarcoma | 38 | 32 | 6 |
| Sarcoma NOS | 7 | 1 | 6 |
| Other sarcoma | 3 | | 3 |
| Benign | 1 | | 1 |
| Not diagnostic | 3 | | 3 |
| Number | 52 | 33 | 19 |

**Table 3.** *Analysis of the 19 atypical osteosarcomas according to radiology and cytology*

| Radiology (19 atypical) | Cytology |
|---|---|
| Typical appearance, but atypical site, 1 | 1 osteosarcoma |
| Atypical appearance, but typical site, 8 | 3 osteosarcoma<br>2 sarcoma NOS<br>1 GCT<br>1 low-grade osteosarcoma<br>1 not diagnostic |
| Both atypical appearance and site, 10 | 2 osteosarcoma<br>4 sarcoma NOS<br>1 PNET<br>1 MFH<br>2 not diagnostic |

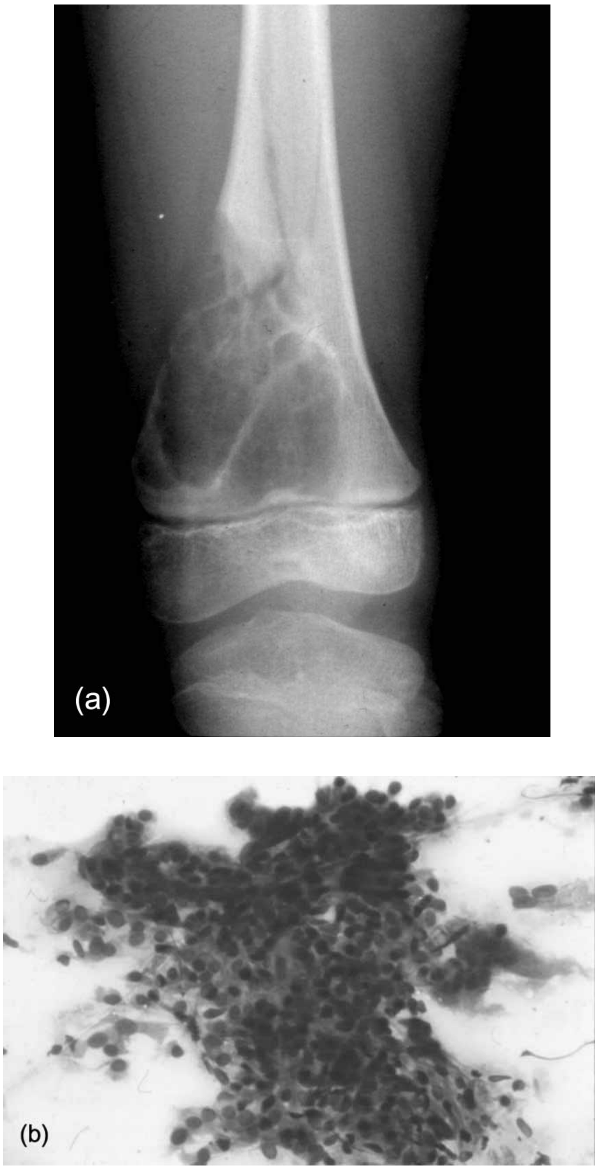

**Fig. 2.** *Atypical high-grade osteosarcoma in a 7-year-old boy. Lesion of the distal femur radiographically suggestive of an aneurysmal bone cyst (a). The cytological smear showed monomorphic hypercromatic cells, which were interpreted to represent a GCT (b).*

by cytology was obtained in six of 19 cases. All six exhibited a mixture of osteoclasts and osteoblasts with atypia and osteoid. The cytological diagnoses were confirmed by histopathology of open (two cases) and core biopsy (two cases). In the remaining two, adjuvant chemotherapy was given based solely on cytology confirmed by postoperative histopathology.

Another six atypical cases showed osteoclasts and osteoblast like cells with varying degree of atypia, but no osteoid, suggesting high-grade mesenchymal malignancy. The OS diagnosis was assessed by histopathology on preoperative open (three cases), core (one case) and postoperative biopsy (two cases). As for the latter two patients, not eligible for the chemotherapy preoperatively because of high age (49 and 67 years), the non-specific sarcoma diagnosis was considered sufficient for decision about treatment, i.e., wide excision.

In the remaining seven radiologically atypical cases, a conclusive cytological diagnosis other than OS was made in four, i.e., three other sarcomas and one benign lesion. One case, a 14-year-old boy with a lesions of distal radius, displayed clinical, cytological and immunocytochemical findings of Ewing's/PNET, although radiology was equivocal (Fig. 3). He was treated preoperatively according to a Ewing's sarcoma protocol including local radiation. Histopathological examination of tissue from the resected specimen suggested an uncommon (6, 7) small-cell OS type, i.e., an epithelioid variant, showing poor response. In another case (male, 78 years) with the cytological diagnosis of low-grade OS, wide excision was performed and high-grade OS was diagnosed on histopathology. The third case (female, 36 years) with the cytological diagnosis of high-grade sarcoma, most likely MFH, underwent open biopsy; histopathology of the excised tissue suggested high-grade OS. The patient died of complications from the preoperative chemotherapy. The fourth case (male, 7 years, Fig. 2) with the cytological diagnosis of giant cell tumour (GCT) underwent curettage; histopathology of the tumour material first suggested aneurysmal bone cyst, but was revised to high-grade OS after external consultation. The patient underwent amputation and the OS diagnosis was confirmed by histopathology of the resected specimen. In the remaining three atypical cases, the aspirates were insufficient for a cytological diagnosis (Tables 1 and 3); the OS diagnosis was made by histopathology of open biopsy.

Taken together, the histopathological diagnosis of the 19 atypical OS cases was associated with considerable difficulties in four, prompting consultation of national as well as international expertise before a consensus diagnosis of OS could be established (Table 4).

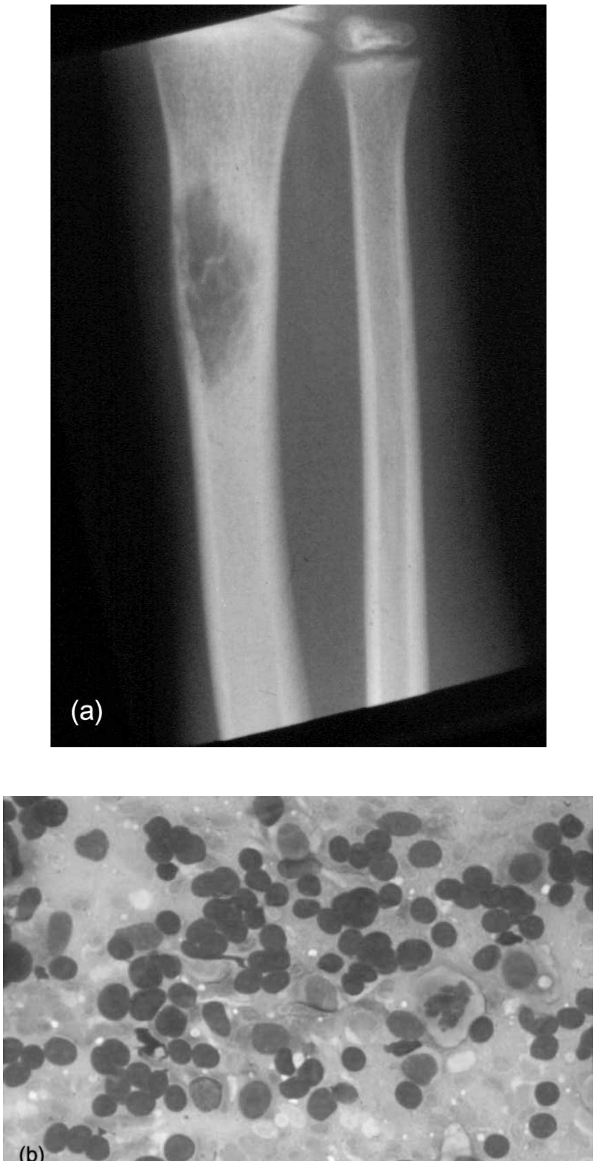

**Fig. 3.** *Atypical osteosarcoma in a 13-year-old boy, with a lesion of the distal radius, with radiographic features suggestive of Ewing's sarcoma, eosinophilic granuloma or osteomyelitis (a). Cytology showed a small cell tumour with features suggesting a Ewing's/PNET (b).*

**Table 4.** *The four cases associated with diagnostic difficulties*

| Age | Site | Radiology | Cytology | Histology | Second opinion | Final diagnosis |
|---|---|---|---|---|---|---|
| 7 | Distal femur | ABC | GCT | ABC? | ABC? | OSG |
| 19 | Proximal humerus | GCT | Primary bone tumour NOS | OSG? | OSG | OSG |
| 36 | Ulna diaphysis | Myeloma | High grade sarcoma | OSG? | OSG | OSG |
| 49 | Distal femur | Chondrosarcoma | High grade sarcoma | OSG? | OSG | OSG |

Of the 19 atypical OS, eight underwent preoperative open biopsy. These eight biopsies and one postoperative specimen were histopathologically reviewed (Mayo Clinic). Seven cases were classified as high-grade OS, whereas two caused diagnostic difficulties. In one case, the distinction between high-grade OS and mesenchymal chondrosarcoma could not be made. Another case was classified as a chondroblastoma. Thus, in two radiologically atypical cases, in which the aspirates failed to provide diagnostic material, histopathology proved to be difficult. The treatment diagnosis high-grade OS was established only after several consultations.

The distribution of the whole series of 52 cases according to cytology and radiology is shown in Table 2.

### Discussion

Our study shows that a combined approach of radiology and cytology provides sufficient information for the diagnosis of high-grade osteosarcoma so as to obviate open biopsy in two-thirds of cases.

It must be emphasised that the present study was retrospective and that the cases were selected according to treatment diagnosis. Thus, the study does not allow assessment of the cytological accuracy in the diagnosis of osteosarcoma in a series of unselected bone lesions. However, it clearly demonstrates the diagnostic strength of concordance between radiology and cytology in the management of high-grade osteosarcoma. This is supported not only by other reports on osteosarcoma,[11,12] but also by studies of unselected series of bone lesions.[13–15] Although we believe that our series only includes true cases of OS, there were two cases, both radiologically atypical, in which the preoperative cytological as well as histopathological diagnosis of osteosarcoma failed to comply with that of one external reviewer, who, notably, was not given any clinical information. Thus, one case was classified as borderline between chondroblastic osteosarcoma and mesenchymal chondrosarcoma and the other as a chondroblastoma. The latter patient with a lesion in thoracic spine died of metastatic disease within 1 year after diagnosis. Apart from these two cases, which we still believe to represent high-grade osteosarcoma, there was full compliance between the treatment and review diagnoses.

Admittedly, the final diagnosis of high-grade osteosarcoma may be questioned in those cases (23 typical and three atypical), in which postoperative histopathology was based on specimens from patients subjected to chemotherapy. Given a good response, acellularity may pose difficulties in recognising osteosarcoma. However, the typical osteoid formed by the tumour cells is not affected by chemotherapy; it remains in areas previously occupied by viable tumour tissue and there may also be scattered cells with significant bizarre nuclei. Thus, despite complete tumour response, osteosarcoma is commonly still recognisable.[16] The histopathological review (Rizzoli Institute) could not identify any single case in which the preoperative cytological diagnosis of high-grade OS should be questioned. As for the remaining 26 cases, there was fresh tissue (no chemotherapy) for histopathological analysis of either preoperative (23 cases) or postoperative (three cases) specimens. Therefore, we are confident that the 52 cases of the present series represent high-grade osteosarcoma, despite the lack of preoperative histopathological specimens in 26 cases and the fact that the OS diagnosis was questioned by one external reviewer in two cases.

In our series, 33 cases of osteosarcomas were radiographically classified as typical and 19 as atypical. The classification used, which considers both appearance and site, should probably be revised. Thus, appearance proved to be a much stronger indicator of the diagnosis than site. There was only one case exhibiting typical appearance, but yet classified as atypical because of atypical site (proximal fibula). Despite the well known anatomical distribution of osteosarcoma in the skeleton,[17,18] it appears that site is a weak diagnostic feature. Therefore, it seems that the radiological diagnosis of osteosarcoma can be confined to appearance. This, however, does not preclude falsely positive and negative cases, which on the other hand can be identified by cytology.[8] Although, the diagnosis of osteosarcoma cannot be exclusively based on a typical radiological appearance, this feature appears to be highly valid when combined with cytology. Thus, the radiographically typical 33 cases were correctly diagnosed preoperatively by cytology, except for one case classified as sarcoma NOS. Although it may be claimed that cytology merely provides confirmatory information, it remains absolutely necessary for decisions on therapy to exclude occasional radiologically osteosarcoma-like lesions.

In radiographically atypical cases, open biopsy should be recommended if cytology fails to give a specific diagnosis. Thus, among the 19 atypical cases, four cytological subgroups apart from osteosarcoma could be identified (Table 1): (1) sarcoma NOS, (2) other sarcoma histotype, (3) falsely benign and (4) non-diagnostic. Open (nine cases) or core biopsy (three cases) was done in 12 of the 19 atypical cases. Yet, the diagnostic difficulties remained in four cases (Table 4). Serious consequences of the diagnostic difficulties pertained to one case, assessed by our pathologist and the external expert as an aneurysmal bone cyst treated by curettage, in which the analysis of the surgical specimen prompted a change of the diagnosis to telangiectatic osteosarcoma and subsequent amputation (NED 8 years follow-up). As for the seven radiologically atypical cases, which did not undergo preoperative open or core biopsy, decision on therapy was based on cytology alone. Because of high age, three of these seven were excluded from preoperative chemotherapy and underwent wide excision. All three received correct treatment according to the final diagnosis of OS. In the remaining four cases, the decision to start preoperative chemotherapy was solely based on an unambiguous cytological diagnosis of osteosarcoma (three cases) and Ewing's sarcoma/PNET (one case). In the latter case, immunocytochemistry corroborated the cytological diagnosis. Yet, the diagnosis was changed, after histopathological analysis of the postoperative material, to small cell OS. Notably, this entity is reported to exhibit not only similar histological features to Ewing's, but also to express the same cytogenetic translocation (chromosome 22q12).[19–22] Nonetheless, the patient received postoperative chemotherapy according to the osteosarcoma protocol (NED 6 years follow-up). On the whole, in our series, it seems that cases causing diagnostic difficulties for the cytologist and radiologist often also cause difficulties for the histopathologist. These cases were evenly distributed over the study period. Hence, it seems that diagnostic difficulties, albeit pertaining to few cases, can be expected regularly. Presumably, the use of complementary methods such as immunocyto-(histo)-chemistry and molecular genetics will prove to be useful in resolving also the difficult cases.

Altogether, cytology offered the correct diagnosis in 38 of 52 cases. Another seven cases were classified as sarcoma NOS, which were primarily managed by surgery (three cases) or open biopsy for the decision about chemotherapy (four cases). The hazard of relying on cytology pertained to the two cases classified as Ewing's sarcoma and giant cell tumour. Notably, both were radiologically atypical. These observations underline the importance of combining cytology and radiology in the diagnosis of high-grade osteosarcoma. Whenever the two methods fail to articulate, open biopsy should be done.

### References

1. Fyfe IS, Henry AP, Mulholland RC. Closed vertebral biopsy. *J Bone Joint Surg (Br)* 1983; 65: 140–3.
2. El-Khoury GY, Terepka RH, Mickelson MR, Rainville KL, Zaleski MS. Fine-needle aspiration biopsy of bone. *J Bone Joint Surg (Am)* 1983; 65: 522–5.
3. Tehranzadeh J, Freiberger RH, Ghelman B. Closed skeletal needle biopsy. *Am J Radiol* 1983; 140: 113–5.
4. Hadju SI, Melmaed MR. Limitations of aspiration cytology in the diagnosis of primary neoplasms. *Acta Cytol* 1984; 28: 337–45.
5. Bhatia A. Problems in the interpretation of bone tumors with fine needle aspiration. *Acta Cytol* 1984; 28: 91–2.
6. Simon MA, Biermann JS. Biopsy of bone and soft tissue lesions. *J Bone Joint Surg (Am)* 1993; 75: 612–21.
7. Huvos AG. The importance of the open surgical biopsy in the diagnosis and treatment of bone and soft tissue tumors. *Hematol Oncol Clin North Am* 1995; 9: 541–4.
8. Kreicbergs A, Bauer CFH, Brosjö O, Lindholm J, Skoog L, Söderlund V. Cytological diagnosis of bone tumours. *J Bone Joint Surg (Br)* 1996; 78: 258–63.
9. Wedin R, Bauer HFC, Lindholm J, Skoog L, Söderlund V. Cytological diagnosis of skeletal lesions: analysis of based on fine needle aspiration biopsy in 110 tumour patients. *J Bone Joint Surg. (Br)* 2000; 82: 673–8.
10. Söderlund V, Tani E, Skoog L, Bauer HFC, Kreicbergs A. Diagnosis of skeletal lymphoma and myeloma by radiology and fine-needle aspiration cytology. *Cytopathology* 2001; 45: 157–67.
11. White VA, Fabnning CV, Ayala AG, Raymond AK, Carrasco CH, Murr JA. Osteosarcoma and the role of fine-needle aspiration. A study of 51 cases. *Cancer* 1988; 62: 1238–46.
12. Kilpatrick SE, Ward WG, Bos GD, Cauvenet AR, Gold SH. The role of fine needle aspiration in the daignosis and management of osteosarcoma. *Pediatr Pathol Mol Med* 2001; 20: 175–87.
13. Jorda M, Rey L, Hanlay A, Ganjei-Azar P. Fine-needle aspiration cytology of bone: accuracy and pitfalls in cytodiagnosis. *Cancer* 2000; 25: 47–54.
14. Ward WG Sr, Kilpatrick S. Fine needle aspiration biopsy of primary bone tumours. *Clin Orthop* 2000; 373: 80–7.
15. Dodd LG, Scully SP, Cothran RL, Harrelsson JM. Utility of fine-needle aspiration in the diagnosis of primary osteosarcoma. *Diagn Cytopathol* 2002; 27: 350–3.
16. Raymond AK, Simms W, Ayala AG. Osteosarcoma. Specimen management following primary chemotherapy. *Hematol Oncol Clin North Am* 1995; 4: 841–67.
17. Dahlin DC, Unni KK. *Bone Tumours*, 4th ed. Springfield, IL: Charles C Thomas, 1986.
18. Jaffe HL. *Tumors and Tumorous Conditions of the Bones and Joints*. Philadelphia, PA: Lea & Febiger, 1958.
19. Bertoni F, Present D, Bacchini P, Pignatti G, Picci P, Campanacci M. The Instituto Rizzoli experience with small cell osteosarcoma. *Cancer* 1989; 64: 2591–9.
20. Picci P. Osteosarcoma and other cancers of bone. *Curr Opin Oncol* 1992; 4: 674–80.
21. Dorfman H, Czerniak B. *Bone Tumors*. St. Louis, MO: Mosby, 1998; 658.
22. Ushigome S, Nakamori K, Nikaido T, Takagi M. Histologic subclassification of steosarcoma: differential diagnostic problems and immunohistochemical aspects. *Cancer Treat Res* 1993; 62: 125–37.
